# Methodological evaluation of the psychometric properties of medication-related quality indicators in residential aged care homes: a systematic review

**DOI:** 10.1093/intqhc/mzag063

**Published:** 2026-05-13

**Authors:** Lakeesha S Liyanage, Mark Naunton, Louise S Cox, Thilini Sudeshika, Gregory M Peterson, Sam Kosari

**Affiliations:** Discipline of Pharmacy, Faculty of Health, University of Canberra, Bruce, ACT 2617, Australia; Discipline of Pharmacy, Faculty of Health, University of Canberra, Bruce, ACT 2617, Australia; Discipline of Pharmacy, Faculty of Health, University of Canberra, Bruce, ACT 2617, Australia; Discipline of Pharmacy, Faculty of Health, University of Canberra, Bruce, ACT 2617, Australia; School of Pharmacy and Pharmacology, University of Tasmania, Sandy Bay, TAS 7005, Australia; School of Health and Biomedical Sciences, RMIT Bundoora West Campus, RMIT University, Bundoora, VIC 3083, Australia

## Abstract

**Background:**

Quality indicators (QIs) are important for tracking and enhancing healthcare quality. In residential aged care homes (RACHs), medication-related QIs help ensure the quality of medication usage and patient care. To ensure the effective use of QIs, it is essential that they are reliable and satisfy other psychometric properties. This study explored the psychometric properties, including validity, reliability, feasibility, applicability, sensitivity, measurability, appropriateness, and usefulness, of medication-related QIs used in RACHs, as well as the methodologies employed in studies evaluating these QIs.

**Methods:**

A systematic search was conducted using CINAHL, MEDLINE, PsycINFO, Scopus, and Web of Science Core Collection, covering studies published since inception until December 2025. Covidence software was used for title and abstract screening and full-text reviewing. Medication-related QIs, psychometric properties evaluated, and the employed methodology were extracted from the studies and were critically analyzed to provide an understanding of current QI evaluation techniques.

**Results:**

The search identified 15 247 studies. After removing duplicates and applying inclusion/exclusion criteria, 21 full-text studies were included, with an average methodological quality score of 60% based on the Mixed Methods Appraisal Tool. Psychotropic drug use and polypharmacy were commonly tested medication-related QIs. Validity (reported in 12 studies), reliability (in 9 studies), and feasibility (in 8 studies) were the most frequently assessed psychometric properties. The Delphi method was the primary approach for validity and feasibility testing.

**Conclusions:**

There were inconsistencies among the methods used for testing the psychometric properties of the QIs. This highlights the need for a standardized protocol for testing medication-related QIs for use in aged care to ensure their reliability and effectiveness.

## Introduction

Residential aged care homes (RACHs), sometimes called nursing homes, cater to older individuals who are unable to reside in their own homes, offering housing along with personal care, nursing, and access to other healthcare services [1]. Residents in RACHs often encounter medication-related challenges. A report from Australia found that 98% of residents in RACHs have at least one medication-related issue [2]. These include polypharmacy, generally due to multiple coexisting medical conditions, and the use of potentially inappropriate medications, which are those that pose a higher risk than clinical benefit [3]. A recent review found that the global prevalence of polypharmacy, defined as the concurrent use of nine or more medications, in adults aged at least 65 years was 45% [4]. Furthermore, an Australian study found that 46% of hospital admissions among aged care residents were due to medication management issues [5].

A quality indicator (QI) is a tool that can be used to measure the quality of care [6]. Assessing QIs helps healthcare providers evaluate and enhance care quality [6]. The literature uses varied terms, including clinical and performance indicators for QI tools, reflecting a lack of a standard definition for these tools despite their wide use and unified objective of enhancing quality. Commonly used QIs in RACHs include the number of falls, unplanned weight loss, pressure injury rates, hospitalization rate, and medication-related QIs [7]. Medication-related QIs have been defined as those designed to measure the safety and quality of medicine use, thereby supporting improvements in clinical practice and resident outcomes [8]. For example, indicators including the prevalence of benzodiazepine, antipsychotic, and opioid use are employed to assess medication safety [9–13].

Some countries have instituted their own data collection programs within RACHs, focusing on QIs [7]. These initiatives allow the harnessing of data to drive enhancements in care quality. However, the effectiveness of these programs is contingent upon the robustness of the data collected, the appropriateness of the QIs used, and the capacity to implement changes based on the insights gained. Ideal QIs are characterized as being specific, sensitive, valid, reliable, well discriminating, and relevant, facilitating useful comparisons among similar settings [14–16]. Although achieving a completely error-free quality measure is impractical, it is essential to thoroughly test QIs for acceptability, feasibility, reliability, validity, and sensitivity to change during both their development and application [17]. This approach enhances their effectiveness in quality improvement strategies as QIs derived from rigorous scientific evidence are more likely to yield effective results [17]. It is essential to evaluate the common criteria, attributes, and characteristics, collectively referred to as the ‘psychometric properties’ of QIs. A systematic review by Hillen *et al.* [18] has emphasized the scientific merit of QIs, considering psychometric properties such as content, face, concurrent and predictive validity, and real-life application, including feasibility and external validity. Various strategies have been employed for the assessment of the psychometric properties of QIs [19–22], but no universal reference is available. The psychometric properties described in the literature are displayed in Table 1.

**Table 1:** Psychometric properties and their definitions.

Psychometric property	Description
Validity	Refers to the degree or extent to which a measure achieves the purpose for which it is intended and is determined [23, 24].
Face validity	If the QI is meaningful and relevant to the key audience it satisfies face validity [25]. It is achieved when an expert/s or researcher/s familiar with the subject matter assesses the instrument and determines that it effectively measures the desired characteristic or trait [26].
Content validity	Content validity refers to how well the items chosen for a measurement instrument accurately represent the variables within the construct they are intended to measure. It assesses the extent to which the items effectively cover the content domain of interest [27].
Construct validity	Construct validity refers to how well an instrument or measurement tool accurately assesses the trait or theoretical concept it is designed to measure. Essentially, construct validity assesses the extent to which a scale or instrument is meaningful and useful in practical applications [24].
Criterion Validity	Criterion-related validity is evaluated when one wants to understand how scores on a test relate to a specific criterion or outcome [24].
Concurrent validity	Concurrent validity is a type of criterion validity that measures how well a new test or measurement correlates with an established, validated test taken simultaneously [28].
Reliability	Reliability is the extent to which a measurement procedure produces the same results on repeated trials [24].
Test–retest reliability	Considered as consistency of scores over time, demonstrating reliability, or stability when repeated testing with the same group of participants yields similar or identical scores [24].
Interrater reliability	Represents the consistency of measurements obtained by different assessors observing the same events [24].
Internal consistency reliability	Concerns the extent to which items on the test or instrument are measuring the same thing [24].
Feasibility	The extent to which a measure can be successfully used or carried out within a given agency or setting [29].
Sensitivity	It refers to the ability to detect differences in the quality of care across facilities [30]. Furthermore, the assessment of the current baseline of the indicator and potential change in baseline at the end of piloting is considered ‘sensitivity to change’ [19].
Applicability	Perception among stakeholders that a given service, practice, or innovation is agreeable or satisfactory [31].
Appropriateness	This refers to how well an innovation or evidence-based practice is perceived to fit, be relevant, or be compatible with a specific practice setting, provider, or consumer. It also pertains to how well the innovation is perceived to address a particular issue or problem [31].
Measurability	This refers to the capability to accurately quantify or assess progress [32, 33].
Usefulness	Usefulness refers to the extent to which these indicators provide valuable and actionable information that can lead to improvements in healthcare quality and outcomes. It measures whether the QIs help healthcare professionals and organizations achieve their goals, such as enhancing patient care, optimizing processes, and ensuring better health outcomes [34].

It is crucial to ensure that measuring and reporting medication-related QIs is feasible. For instance, conducting thorough medication reviews to identify potentially inappropriate medications may be time-consuming, thereby reducing the feasibility of this measurement approach for routine use as a QI [35]. Although previous systematic reviews have provided comprehensive information on QIs used in aged care, none have synthesized the methodologies employed to assess the psychometric properties of medication-related QIs, which was the focus of this article. Therefore, this systematic review examined the psychometric properties evaluated for medication-related QIs in RACHs and assessed the methods used to test them.

## Materials and methods

The protocol for this systematic review was registered in the International Prospective Register of Systematic Reviews (PROSPERO) on 24 December 2023 (Registration No: CRD42023492709 available from: https://www.crd.york.ac.uk/prospero/display_record.php?ID=CRD42023492709). The presentation of this systematic review aligns with the Preferred Reporting Items for Systematic Reviews and Meta-Analyses (PRISMA) checklist, as mentioned by Liberati *et al.* [36].

A comprehensive and systematic search was conducted using CINAHL, MEDLINE, PsycINFO, Scopus, and Web of Science Core Collection databases covering studies published since inception until 30 December 2025. Keywords used in the database search were developed following a scoping review and with assistance from a research librarian (Supplementary Table S1). Original research studies with full articles that reported medication-related QIs in RACHs and tested psychometric properties, such as validity, reliability, sensitivity, feasibility, applicability, appropriateness, usefulness, or measurability, were included. The search was limited to literature in the English language and studies that were conducted in settings other than RACHs (e.g. hospitals, community or disability centres), and review articles were excluded.

Covidence software was used for title and abstract screening, full-text review, and data extraction processes [37]. Citation screening was conducted to include relevant studies and to ensure comprehensive coverage of relevant literature. When additional information on methodology was needed, direct communication with authors was initiated via email; if the authors did not respond, these studies were excluded from the review.

Two reviewers (LL and TS) independently applied the specified inclusion and exclusion criteria to the titles and abstracts to 15% of all the initial search results for this review. Inter-rater reliability was calculated for the first 10% of studies. Then inter-rater reliability was re-evaluated for an additional 5% of studies, and after reaching 100% agreement among both researchers, the first researcher continued screening the remaining 85% of the studies. This approach has been previously followed in published systematic reviews [38, 39]. Full-text review of the studies remaining following the initial screening was independently conducted by two reviewers (LL and LC), and discrepancies were resolved in consultation with a third researcher (SK).

The process of data extraction and synthesis comprised multiple phases. Initially, one author (LL) extracted descriptive attributes of the included studies, focusing on QIs and their psychometric properties. Subsequently, stepwise methodologies pertaining to each psychometric property, as outlined in the studies, were extracted. In instances of ambiguity or insufficient details, authors were contacted via email for clarification. The second researcher (LC) reviewed the extracted data to ensure its accuracy. Further, the extracted data were discussed with two other researchers (SK and MN). Qualitative thematic synthesis was employed to combine and assess the extracted data [40].

The evaluation of the methodological quality of the studies selected was independently conducted by two researchers (LL and LC/TS) using the Mixed Method Appraisal Tool (MMAT) [41]. Following the identification of each article’s study design, an assessment was made based on five criteria. The MMAT scores of the included studies have been presented as the number of Yes, No, and Can’t tell answers for each MMAT criterion. Consensus regarding quality scores was reached through deliberation between the two reviewers [41].

## Results

**Figure 1:**
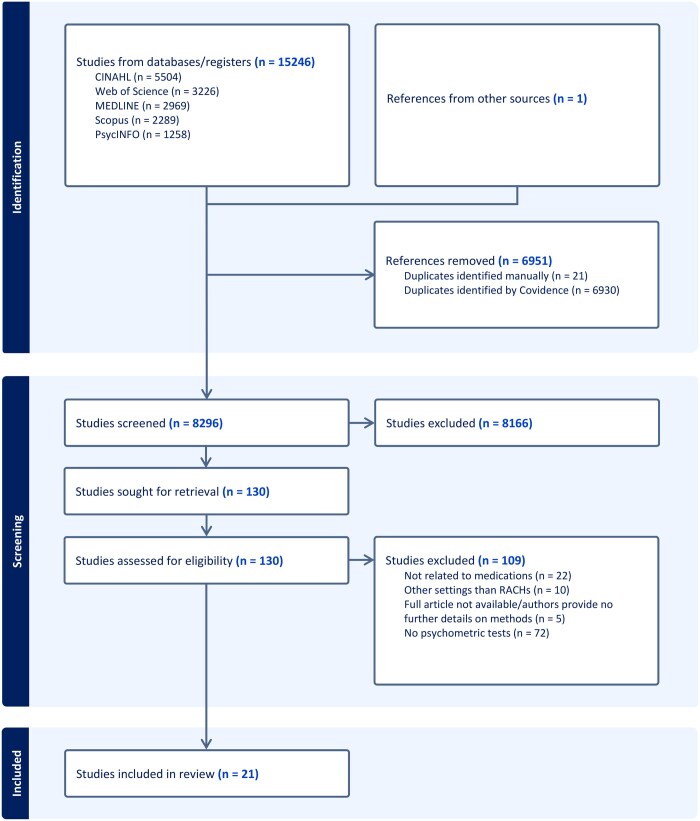
PRISMA flow chart. This diagram outlines the number of records identified, screened, assessed for eligibility, and included in the final review.

The search yielded a total of 15 247 studies, comprising 15 246 identified via database searches and 1 sourced from manual examination of references cited in retrieved articles and pertinent systematic reviews. Following the elimination of duplicates (*n* = 6951) and the evaluation of studies against the inclusion and exclusion criteria (*n* = 8296), 130 studies were included for full-text review. After full-text review, 21 were found suitable for inclusion in the review. Figure 1 illustrates the study’s selection process.

For the title and abstract screen, there was 6.2% disagreement for the initial 775 studies, before consensus was reached among the two reviewers. To confirm the agreement, a further 388 studies were screened for title and abstract among the included studies. After this round, there was 100% agreement among the two reviewers. For the full-text reviews, 100% agreement was reached between two reviewers.

The structured abstracted data were categorized as follows: name of the author, year of publication, country, medication-related QI, numerator/denominator or description of QI, and psychometric properties and other properties (Supplementary Table S3). The quality of each study, as assessed using MMAT, is presented in Supplementary Table S2. A higher count of ‘Yes’ indicates superior quality [41, 42]. The quality of the included studies varied, but they were all evaluated for their psychometric properties and methodologies due to their potential to enhance understanding in this research area.

A summary of the medication-related QIs and psychometric properties assessed in the 21 included studies is presented in Table 2. The methodological evaluation of the psychometric tests in the included studies is shown in Table 3. Medicines commonly used for evaluation included antipsychotics, antibiotics, opioids, benzodiazepines, anxiolytics, and antidepressants, as well as measures of polypharmacy. In addition, medication-related processes, including medication reviews and hospitalizations, were also used as QIs (Supplementary Table S3). QIs related to psychotropic medications were the most frequently assessed QIs. Among these QIs, seven studies (33%) evaluated the validity [9, 11, 12, 43–46], five studies (24%) evaluated feasibility [13, 22, 43, 44, 46], and four studies (19%) evaluated reliability [9, 11, 47, 48]. Psychometric properties of the polypharmacy QI were assessed by four studies (19%), with two focusing on reliability [21, 48], one focusing on validity [45], and one focusing on feasibility [22]. Four studies evaluated opioid and pain medications, assessing validity, reliability, and feasibility [10, 13, 46, 49].

**Table 2:** Summary of medication-related QIs and psychometric properties assessed by the included studies.

Quality Indicator (*n*)	Validity (*n*)	Reliability (*n*)	Feasibility (*n*)	Sensitivity (*n*)	Applicability (*n*)	Measurability (*n*)	Appropriateness (*n*)	Usefulness (*n*)
Psychotropic medications (12)	7	4	5	1	–	–	1	1
Polypharmacy (4)	1	2	1	–	–	–	–	–
Opioid and pain medications (4)	3	2	2	–	–	–	–	1
Antibiotics (2)	1	–	1	–	1	1	–	–
Medication reviews and medication appropriateness (4)	3	1	2	–	–	–	1	–
Cardiovascular medications (1)	1	–	1	–	–	–	–	–
Diabetes medication management (1)	1	–	1	–	–	–	1	–
Medication monitoring (1)	1	–	1	–	–	–	1	–
Providing information to resident/family (1)	1	–	1	–	–	–	1	–
No. of patients with hospitalization/ED visit (1)	–	–	1	–	–	–	–	1
No. of patients with anti-dementia medications (2)	1	–	2	–	–	–	–	1
New medications received (3)	2	1	2	–	–	–	1	–
Number of days injections of any type received (1)	–	1	–	–	–	–	–	–
Medication with anticholinergic effects (1)	1	–	1	–	–	–	–	–
PPIs (1)	1	–	1	–	–	–	–	–
Discharge from a hospital to a RACH (2)	1	1	2	–	–	–	–	–
Influenza vaccine coverage (1)	–	–	–	–	1	1	–	–

ED: emergency department, *n*: number of studies, PPI: proton pump inhibitor, RACH: residential aged care home.

**Table 3:** Methodological evaluation of the psychometric properties of medication-related QI in RACHs.

No.	Medication-related QIs and study	Type of psychometric property	Methodologies used to assess the QIs
1	Antibiotics Asquier-Khati *et al.* [50]	Validity Feasibility	Delphi panel with 2 rounds; structured criteria were given but not provided in the study. Participants: *n* = 20: AMS physicians (*n* = 5), RACH physicians (*n* = 4), infectious diseases specialists (*n* = 3), geriatrician (*n* = 2), infection control specialists (*n* = 2), policymakers (*n* = 2), GP (*n* = 1), pharmacist (*n* = 1) Participants were asked to give their opinion regarding possible uses of indicators in terms of: Making comparisons between RACHs Designing of AMS programs at the facility level Providing feedback to RACH prescribers Public reporting of facility level indicators
2	Medication review, Providing information to residents and families, Medication monitoring and Medication use (refer to Supplementary Table S3 for more details) Hibbert *et al.* [44]	Validity Feasibility Appropriateness	Delphi panel with two rounds; structured criteria were given for indicator feasibility and appropriateness but not for validity. Participants: *n* = 41: representing nursing, research, medicine, speech pathology, optometry, dietetics, physiotherapy, dental, pharmacy, psychology, audiology Participants were asked to rate the below statements against each QI: More eligibility criteria may reduce feasibility, as fewer patients qualify for the indicator Compliance is best assessed at admission or within 90 days Likely to be documented in the RACH record, e.g. indicators associated with lifestyle or exercise advice are less likely to be documented Participants were asked to rate the below statement against each QI: The expected health benefit exceeds the expected negative consequences by a sufficiently wide margin that the procedure is worth doing, exclusive of cost.
3	Polypharmacy No Pharmacy review Chemical Restraints Courtney *et al.* [45]	Face validity Content Validity	Delphi panel with 2 rounds; structured criteria were given for face and content validity. Participants: *n* = 6, including experienced aged care clinicians, managers and researchers, as well as a consumer representative **Face Validity**; Participants were asked to comment on the overall suitability of each QI for use within RACH. **Content Validity**; Participants were asked to comment on the below statements against each QI: The suitability of the assessment question(s) for inclusion, 2. The wording of the assessment question(s), 3. Whether you believe anything should be added
4	Prevalence of antipsychotic medications without a diagnosis of psychosis Xu *et al.* [11]	Construct validity	Method used: Exploratory factor analyses. Domains were identified using principal component methods and refined using the scree plot. QIs were assigned to the best-fitting domain based on factor loadings. Cronbach’s alpha checked reliability, and quarterly sensitivity analyses ensured the domains stayed relevant.
5	19 medication-related QIs (refer to Supplementary Table S3 for more details) Mays *et al.* [46]	Validity Feasibility of implementation	Delphi panel with two rounds; structured criteria were given for the validity and feasibility of implementation. Participants: *n* = 11, experts were selected based on their knowledge and leadership in post-acute and long-term care; professions: medicine (*n* = 9), researcher (*n* = 1), nurse practitioner (*n* = 1). Validity; participants were asked to rate QIs considering the following statements, QI is clear and explicit Adequate scientific evidence or professional consensus supports a strong link between the performance of specified care and outcomes. Improved quality of life is considered an outcome A provider with significantly higher rates of adherence to an indicator would be considered a higher quality Most factors influencing adherence should be within the influence of primary care provider or influenced by external factors, like care givers. Participants were asked to rate QIs for feasibility of implementation, based on staffing resources, physician resources, and expense.
6	Percent of residents on antipsychotics without a diagnosis of psychosis Jones *et al.* [8]	Validity	Method used: A regression model An expert panel identified preventive (anticipatory) and responsive (reactive) strategies as covariates. Principal components analysis extracted 10 uncorrelated preventive and 10 responsive summary variables. Three regression models were run: one with preventive components, one with responsive components, and one with both. The results classified QIs into top, mid, and not valid levels based on their presumed validity.
		Interrater reliability	Reliability among nurse researchers was assessed by having nurses complete two paired assessments and medical reviews with their partner per facility. Kappa coefficient was calculated to determine the level of agreement between the assessors.
7	ACOVE-3—set of QIs: Domain medication use (24 QIs)—(no further information provided) Wenger *et al.* [51]	Validity	Delphi panel with two rounds; structured criteria were given, but not provided in the study. Participants: *n* = 12, Multidisciplinary group (professional backgrounds were not provided)
8	Pain medication appropriateness scale (PMAS) Hutt *et al.* [9]	Content validity	Delphi panel, number of rounds: not provided; structured criteria were given, but not provided in the study. Participants: *n* = 5, pharmacists (*n* = 2), geriatrician (*n* = 1), nurse practitioners (*n* = 2)
		Construct validity	Construct validity was assessed by hypothesizing that residents who denied being in pain would have higher PMAS scores than those in pain. Pilot test was used for this purpose.
		Interrater reliability	A research team member accompanied each research assistant on their first visits to nursing homes. They interviewed residents, observed for nonverbal pain indicators, and reviewed medical records. Interrater reliability was measured by comparing the agreement between the research assistant and the research team member.
		Test–retest reliability	Residents with multiple assessments were studied over a 3-month interval. Correlations between their PMAS scores from two baseline data collection rounds were analyzed. This interval was chosen for expected stability in chronic pain responses. Additionally, correlations were computed at the facility level to reduce the impact of individual healthcare changes over time.
9	(refer to Supplementary Table S3 for more details on QIs) Kröger *et al.* [52]	Face and content validity	Delphi panel with two rounds Participants: *n *= 33, medicine or geriatric medicine (*n* = 9), nursing (*n* = 6), occupational therapy (*n* = 3), psychology (*n* = 3), neuropsychology (*n* = 2), pharmacy (*n* = 4), nutrition (*n* = 3), social work (*n* = 3) Structured criteria were given as below, Scientific evidence for a link between process and outcome Clinical relevance to the care of vulnerable older adults Ability to discriminate between a high- and a low-quality provider Necessity to document this indicator in the patient’s medical record
		Interrater reliability	Interrater agreement was assessed by both observers (study nurses) at least once for the same patient. An overall Kappa statistic was calculated for the agreement between the two observers.
		Feasibility	In a pilot test, it was checked whether the information required for the indicator was present in the patients’ file (*n* = 29 patients).
10	QIs related to medication use and antipsychotic use Hawes *et al.* [10]	Face validity	Face validity was established with debriefing of facility staff. (More details about the methodology were not provided.)
		Interrater reliability	Inter-rater reliability was assessed using facility and research nurses. (More details about the methodology were not provided)
11	(Refer to Supplementary Table S3 for more details) Zimmerman *et al.* [43]	Face validity Concurrent validity	Two validation team members conducted 20 validation studies in 5 states. QIs were assessed through a review of approximately 25 individual resident cases, using a combination of resident observation, resident and staff interviews, and record review. This validation study was designed to answer the following questions about QIs. Are the data and algorithms used to construct the QIs accurate? Does the QI correctly indicate a problem with the quality of care at the resident level (i.e. for the specific resident in the case being investigated)? Is the problem severe enough to suggest a facility-wide problem? Are the problems identified, either on the basis of severity or scope, at a level that would warrant the citation of a deficiency under Federal regulations? **Concurrent validity** ; A validation team and a survey team conducted pilot test surveys. The validation team assessed the pre-selected QIs on the basis of facility QI reports. Those were assessed using approximately 25 individual resident cases. The survey team conducted the regularly scheduled survey and results were compared.
		Feasibility	Survey with telephone calls. Asked staff from RACHs in terms of easy to interpret and integrate the QI.
12	Polypharmacy Favez *et al.* [21]	Interrater reliability	The data were collected for a minimum of 6 months in 152 nursing homes by the researchers. Intraclass 2 (ICC2) correlation was computed. ICC2 is the ratio of group variance to total variance/*k*, where *k* is the number of nursing homes, and VG is the group variance. i.e. ICC2 = VG/((VG + π^2^/3) × (1/*k*)) The higher a QI’s ICC1, the higher its ICC2. The ICC2 typically ranges from 0.6 to 1.0, with values closer to 1 indicating higher measurement reliability.
13	Days received—antipsychotics Mor *et al.* [47]	Interrater reliability	Two research nurses collected data at each facility, and their assessments were pooled to check overall reliability due to a lack of individual cases. This pooling established the nurses as a reliable ‘gold standard’. QIs based on their ratings were compared with those from facility nurses. The Kappa statistic and percentage agreement were used to compare the assessments of facility nurses with the ‘gold standard’ research nurses.
14	Number of medications New medications Injections Days received: Antipsychotics Antianxiety/hypnotics Antidepressants Morris *et al.* [48]	Interrater reliability	Three methods were used. Percentage of item agreement was calculated as the number of pairs of assessments of an item on the same resident that were the same, expressed as a percentage of all pairs in the reliability comparison sample. Measures of association, or correlation, used Phi for dichotomous indicators and Rho for numerical statistics to describe the matching and mismatching of assessment pairs. Measures of congruence: The Spearman Brown intraclass correlation coefficient measured agreement between facility and project nurses. An MDS item with a value of 0.40 or higher was considered minimally reliable.
15	Medication Appropriateness Index Stuijt *et al.* [57]	Interrater Reliability	Each patient was assessed twice by two independent reviewers. Inter-rater reliability was determined by comparing their scores using kappa statistics. Kappa values of 0.41–0.60 indicate moderate reliability, 0.61–0.80 indicate substantial reliability, and values above 0.81 indicate excellent reliability.
16	Percentage of residents prescribed ≥9 medications (not including topical, dietary supplements, short term or PRN medications) Percentage of residents who received antipsychotic medications Inacio *et al.* [22]	Feasibility	Five expert researchers and clinicians assessed and rated each indicator’s feasibility. They used a scale from 1 (does not meet criteria) to 9 (meets criteria perfectly) for their evaluations using the below criteria, Attribution: The level of attribution specified in measures is appropriate (measure ties the outcomes to the appropriate unit of the analysis) and is clearly stated. Providers control: the performance measure addresses an intervention that is under the influence of the providers being assessed. Usability: the results of the measure provide information that will help the provider improve care. Burden: data collection is feasible, and the burden is acceptable (low, moderate or high).
17	Ten medication-related QIs Jennifer G. Burgess [12]	Feasibility Usefulness	Delphi panel with two rounds; structured criteria were given for feasibility and usefulness. Participants: *n* = 10, experts included geriatric psychiatry, geropsychology, geriatric medicine, nursing, pharmacy, and research. Participants were asked to rate each QI against the following statements. The data necessary to calculate the measure is readily available The data necessary to calculate the measure is likely reliable The data necessary to calculate the measure is likely unbiased Usefulness: Participants were asked to rate each QI against following statements. The measure could have a substantial impact on clinical care You would likely use or encourage incorporating this measure for quality improvement in your practice The numerator includes a clinically significant population.
18	Four medication-related QIs Bell *et al.* [58]	Feasibility	Delphi panel with two rounds; structured criteria were given but not provided in the study. Participants: *n* = 10, nurses, physicians, pharmacists, policy makers and academic researchers
19	Antipsychotic use without psychosis Estabrooks *et al.* [59]	Practice sensitivity	Delphi panel, number of rounds: not provided; structured criteria were given. Participants: *n* = 16, practicing physicians (*n* = 4), nurses (*n* = 8), and decision/policymakers (*n* = 4) Rank the items on the list for overall ‘practice sensitivity’ Identify the domain to which the QI was most sensitive (nursing care, physician care, or policy maker)
20	Eleven PI (appropriateness of prescription) for antibiotics (refer to Supplementary Table S3 for more details) Simon *et al.* [60]	Applicability	If a PI was relevant to at least 10 clinical situations in a RACH, it was considered applicable. PI scores couldn’t be calculated if there were fewer than 10 prescriptions for suboptimal practices (drugs that shouldn’t be prescribed) or if there were fewer than 10 prescriptions for either the numerator or denominator for other PIs describing both suboptimal and good practices. Overall, a PI was applicable if it could be calculated for more than 75% of the nursing homes.
		Measurability	Data were collected from the RACHs (reimbursement database of the Regional Health Insurance Fund, France). If data were available for at least 75% of RACHs, they were considered as measurable.
21	Appropriate use of opioids in long-term care Resnick *et al.* [49]	Validity Interrater reliability	Pain was evaluated based on the minimum data set (MDS) verbal response item: Have you had any pain or hurting at any time in the last 5 days? Data 137 recruited residents from 6 nursing home communities; pain was evaluated based on the MDS. The assessment of appropriate use of opioids includes 10 items. Validity—Rasch analysis to check linear probabilistic relationship between a person’s ability and the item’s difficulty; INFIT and OUTFIT statistics to assess item fit. Reliability was tested in two ways. First, Rasch analysis was used to see how accurately each item reflected the underlying trait being measured. This included calculating an item separation index, where a value of at least 0.7 indicated that the items were consistently measuring the same concept. Second, the consistency of the scoring was measured between two independent evaluators by calculating a correlation between their scores, a higher correlation meant that different raters produced similar results, showing good score consistency.

AMS: anti-microbial stewardship, GP: general practitioners, ICC: intra-class correlation, INFIT: information-weighted fit, OUTFIT: outlier-sensitive fit, QI: quality indicator, VG: group variance, MDS: minimum data set, PI: proxy indicator, PMAS: Pain Medication Appropriateness Scale, PRN: pro re nata (as needed), RACH: residential aged care home.

Overall, validity assessment of medication-related QIs was reported in 12 studies (57%) [9–12, 44–46, 49–52]. Among these studies, only three met 80% or more of the criteria in the MMAT assessment. Among the 12 studies, 4 studies (33%) conducted face validity assessment [11, 43, 45, 52], 3 studies (25%) assessed content validity [10, 45, 52], 2 studies (17%) evaluated construct validity [10, 12], and 1 study assessed concurrent validity [43]. Construct validity was assessed using pilot and exploratory factor analysis methods in two QIs (prevalence of antipsychotic medications without a diagnosis of psychosis and pain medication appropriateness scale) in two studies [10, 12]. Both of those studies satisfied 80% of the quality criteria in the quality assessment using MMAT. Similarly, concurrent validity assessment, which is a type of criterion validity, was found in one study [43]. Specific types of validity were not specified in six studies (50%) [9, 44, 46, 49–51]. Seven studies (58%) [10, 44–46, 50–52] employed the Delphi method for validity studies, including face and content validity.

There is consensus in the literature that Delphi studies can generally be carried out in two to three rounds [53]. The included studies used expert panels ranging from 5 to 41 members (median 12). Smaller panels in some studies raise concerns about the reliability and validity of their findings. Panel size and areas of expertise varied considerably, and although most studies used multidisciplinary experts, one did not report the professional backgrounds of its panel. Definitions of ‘expertise’ were also broad and inconsistently applied across studies [54]. The commonly accepted criteria for joining an expert panel include having relevant experience and knowledge, being willing and able to participate, having the time to commit, and possessing good communication skills [55, 56]. In several studies, researchers provided structured questions to the Delphi panels, ensuring a consistent approach. However, in other studies, no such structured questions were evident, again highlighting variation in methodologies [10, 50, 51].

Reliability testing of QIs in the context of medication management was reported in nine studies (43%) [9–11, 21, 47–49, 52, 57]. Among these studies, three studies met 80% or more of the criteria. All the studies conducted inter-rater reliability, and only one study conducted test-retest reliability [10].

The feasibility of QIs was evaluated in eight studies (38%) [13, 22, 43, 44, 46, 50, 52, 58]. The average quality score of those studies was 51% in the MMAT assessment, which indicates a moderate level of methodological rigor. Methods used included the Delphi technique [13, 44, 46, 58], surveys [22, 43, 50], and a pilot study [52]. The criteria for feasibility were inconsistent across studies, but commonly included the availability of human resources, associated expenses, time required, data availability and reliability, potential to improve care, and impartiality.

Individual assessments of applicability, practice sensitivity, measurability, appropriateness, and usefulness of QIs were each addressed in separate studies [13, 44, 59, 60]. The studies that discussed applicability and measurability met 80% or more criteria in the MMAT assessment. The studies that discussed practice sensitivity, appropriateness, and usefulness met only 40% or less of the criteria in the MMAT assessment. These assessments have been less frequently evaluated for medication-related QIs in RACHs. The Delphi method was used to assess usefulness and appropriateness, with clear criteria provided for participants to rate these properties [13, 44]. Applicability and measurability were evaluated in one study using a quantitative approach [60].

Additionally, the review identified other assessment properties (Supplementary Table S4), including actionability, importance, between-provider variability, impact, relevance, potential for improvement, specification, clinical evidence basis, and appropriateness of care [12, 13, 21, 22, 44, 60].

## Discussion

### Statement of principal findings

The review found that medication-related QIs used in RACHs have been evaluated with a wide range of psychometric and assessment properties, demonstrating considerable variation in how their quality and performance are measured, noting that the number of studies remains limited. The QI on psychotropic use (*n* = 12) was the most extensively evaluated medication-related QI, while the polypharmacy indicator requires clearer and more consistent definitions to ensure meaningful assessment. For example, this includes specifying whether the focus is on regular, short-course, or PRN (as-needed) medications, and whether the selection is based on prescribed or administered medicines [61].

Validity assessment of medication-related QIs in residential aged care was common, but the methods used, especially Delphi processes, varied widely in rigor, panel composition, and structure, raising concerns about consistency and reliability. Construct and concurrent validity were rarely applied, highlighting the need for more comprehensive and consistent validity testing in future research.

Reliability testing was the second most evaluated psychometric property in the included studies, yet the scope of testing remains limited. Strengthening reliability assessment is essential, as consistent QIs are critical for supporting accurate reporting, informed decision-making, and effective quality improvement in aged care settings [23].

Studies that assessed the feasibility of medication-related QIs in RACHs showed variable methods and inconsistent criteria, but collectively highlighted that feasibility depends heavily on available resources, data accessibility, and the practicality of implementing QIs within routine care.

The concept of sensitivity in QIs is crucial, as it reflects the ability to detect meaningful changes over time, from baseline assessment to the completion of the clinical intervention, a characteristic commonly referred to as sensitivity to change [19, 62, 63]. QIs that are highly sensitive to clinical and policy interventions play a pivotal role in RACHs by enabling early detection of potential quality deficits [59]. This early identification is essential for implementing timely interventions, preventing adverse health outcomes, and continuously improving care standards [14].

The methodological quality of the included studies averaged 60%, with scores spanning from 20% to 100%. Such variability suggests that while some studies demonstrated strong methodological rigor, others had notable limitations, suggesting the need for more robust methodological approaches in future research on medication-related QIs.

### Strengths and limitations

This review employed a comprehensive search strategy that included a wide range of databases. However, most of the QIs identified and included in this systematic review were not comprehensively assessed for all the psychometric properties. Grey literature was not included, which may have led to missing relevant QIs or approaches commonly reported in government and organizational documents. Finally, only studies published in English were included.

### Interpretation within the context of the wider literature

This systematic review reflects broader trends in aged care literature, revealing both advances and persistent gaps in the evaluation of medication-related QIs. Psychotropic use and polypharmacy remain the most commonly targeted areas, consistent with prior studies highlighting their clinical relevance in aged care settings [58]. Validity and reliability were frequently assessed, often using the Delphi method, although methodological variations reflect limitations similar to those reported in earlier reviews [64]. Less frequently examined properties, including sensitivity, applicability, and usefulness, highlight an ongoing challenge of ensuring that QIs are both meaningful and implementable in practice [65]. These findings reinforce calls for more comprehensive standardized frameworks to enhance both methodological rigour and practical relevance in QI assessment in residential aged care settings [66].

### Implications for policy, practice, and research

Ensuring that QIs are technically sound is crucial in aged care, enabling practitioners, policymakers, and organizations to make informed decisions and drive meaningful improvements in care. Methodological inconsistency and limited evaluation of key psychometric properties in studies highlight the need for more standardized medication-related QIs in residential aged care. The limited assessment of psychometric properties for medication-related QIs reduces confidence in whether these indicators accurately reflect medication safety or appropriateness in RACHs. Without evidence of validity and reliability, QIs may lead to misinterpretation of care quality or misdirected improvement efforts. Several factors may explain why psychometric properties have been less frequently evaluated for medication-related QIs in RACHs. Medication-related indicators often require detailed clinical data [67], which may not be routinely collected or easily accessible in many aged care settings. Many RACHs lack integrated electronic medication records, limiting the ability to test data accuracy or reliability. Additionally, many QIs are adopted from other healthcare contexts without formal validation in RACH populations, contributing to gaps in psychometric evidence [23]. Future research should expand the evaluation of infrequently assessed QI psychometric properties.

## Conclusion

This review systematically synthesized and examined methodologies used to test medication-related QIs in RACHs, and identified inconsistencies in the assessment of their psychometric properties. It highlights the need for standardized testing methods and improved reporting practices to enhance QI evaluation.

While validity, feasibility, and reliability were commonly assessed, other psychometric properties, such as sensitivity, usefulness, appropriateness, applicability, and measurability, were assessed and reported less frequently. Key criteria that were not consistently assessed for feasibility included accessibility to data in RACHs, the time and expertise required for QI assessment and reporting, and the simplicity of reporting processes. These factors should be considered to ensure the successful integration of QIs into routine practice in aged care settings. Given the limited number of studies for each psychometric test and the variation in the methodological quality assessment scores, further research is warranted.

## Supplementary Material

mzag063_Supplementary_Data

## Data Availability

The data underlying this article are available in the article and in the supplementary material.
