# Supplementary material for: Methodological evaluation of the psychometric properties of medication-related quality indicators in residential aged care homes: a systematic review
Source: Int J Qual Health Care. 2026 May 13;38(2):mzag063. doi: 10.1093/intqhc/mzag063 (PMC13198374; doi:10.1093/intqhc/mzag063)
Supplement: mzag063_Supplementary_Data [file mzag063_supplementary_data.docx]

**Supplementary table** **1:** **Keywords for the database searches**

| Database: Cumulative Index of Nursing and Allied Health Literature (CINAHL) (via EBSCOhost) | | | | |  |
| --- | --- | --- | --- | --- | --- |
| Search # | | Concept | Search Terms | |  |
| #1 | | Aged care centres | “Aged care facilit*” OR “Care home*” OR “Elderly care” OR “Elderly home*” OR (MH “Gerontologic Care”) OR “Gerontolog* care” OR (MH "Housing for older person*") OR “Home* for the aged” OR (MH “Long term care”) OR “Long term care” OR “Nursing facilit*” OR (MH “Nursing homes+”) OR “Nursing home*” OR “Residential aged care” OR “Residential care” OR (MH “Senior centers”) OR “Senior cent*” OR “Senior home*” | |  |
| #2 | | Psychometric properties | "Applicab*" OR "Feasib*" OR "Reliab*" OR "Sensitive*" OR "Sensitivity" OR "Valid*" OR "Appropriateness" OR "Measurability" OR "Usefulness" | |  |
| #3 | | Quality indicators | "Assessment tool*" OR (MH "Benchmarking") OR “Benchmarking” OR "Benchmark criteria*" OR “Care quality” OR "Care performance indicator*" OR (MH "Clinical Indicators") OR “Clinical indicators” OR “Evaluation indicator*” OR "Key performance indicator*" OR (MH “Outcomes (Health Care)+”) OR “Outcome measure*” OR (MH “Outcome assessment”) OR “Outcome assessment*” OR “Performance assessment*” OR (MH "Process Assessment (Health Care)+") OR “Process Assessment (Health Care)” OR "Performance indicator*" OR "Performance measure*" OR “performance metric*” OR (MH “Quality assessment+”) OR “Quality assessment” OR (MH “Quality assurance+”) OR “Quality assurance*” OR "Quality assessment indicator*" OR "Quality assurance measure*" OR “Quality of care” OR “Quality of health care” OR “Quality of nursing care” OR “Quality indicator*” OR “Quality measure*” OR “Quality metric*” OR "Quality monitoring indicator*" OR (MH “Quality improvement”) OR “Quality improvement” OR "Quality improvement indicator*” OR “Quality standard*" OR “Resident outcome*” OR “Patient outcome” | |  |
| Database: Medline (via EBSCOhost) | | | | |  |
| Search # | | Concept | | Search Terms |  |
| #1 | | Aged care centres | | “Aged care facilit*” OR “Care home*” OR “Elderly care” OR “Elderly home*” OR (MH “Gerontologic Care”) OR “Gerontolog* care” OR (MH "Housing for older person*") OR “Home* for the aged” OR (MH “Long term care”) OR “Long term care” OR “Nursing facilit*” OR (MH “Nursing homes+”) OR “Nursing home*” OR “Residential aged care” OR “Residential care” OR (MH “Senior cent*”) OR “Senior cent*” OR “Senior home*” |  |
| #2 | | Psychometric properties | | "Applicab*" OR "Feasib*" OR "Reliab*" OR "Sensitive*" OR "Sensitivity" OR "Valid*" OR "Appropriateness" OR "Measurability" OR "Usefulness" |  |
| #3 | | Quality indicators | | "Assessment tool*" OR (MH "Benchmarking") OR “Benchmarking” OR "Benchmark criteria*" OR “Care quality” OR "Care performance indicator*" OR (MH "Clinical Indicators") OR “Clinical indicators” OR “Evaluation indicator*” OR "Key performance indicator*" OR (MH “Outcomes (Health Care)+”) OR “Outcome measure*” OR (MH “Outcome assessment”) OR “Outcome assessment*” OR “Performance assessment*” OR (MH "Process Assessment (Health Care)+") OR “Process Assessment (Health Care)” OR "Performance indicator*" OR "Performance measure*" OR “performance metric” OR (MH “Quality assessment+”) OR “Quality assessment” OR (MH “Quality assurance+”) OR “Quality assurance*” OR "Quality assessment indicator*" OR "Quality assurance measure*" OR “Quality of care” OR “Quality of health care” OR “Quality of nursing care” OR “Quality indicator*” OR “Quality measure*” OR “Quality metric*” R "Quality monitoring indicator*" OR (MH “Quality improvement”) OR “Quality improvement” OR "Quality improvement indicator* OR "Quality standard*" OR “Resident outcome*” OR “Patient outcome” |  |
| Database: PsycINFO (via EBSCOhost) | | | | | |
| Search # | Concept | | | Search Terms | |
| #1 | Aged care centres | | | “Aged care facilit*” OR “Care home*” OR “Elderly care” OR “Elderly home*” OR “Gerontolog* care” OR "Housing for older person*" OR “Home* for the aged” OR “Long term care” OR “Nursing facilit*” OR “Nursing home*” OR “Residential aged care” OR “Residential care” OR “Senior cent*” OR “Senior home*” | |
| #2 | Psychometric properties | | | "Applicab*" OR "Feasib*" OR "Reliab*" OR "Sensitive*" OR "Sensitivity" OR "Valid*" OR "Appropriateness" OR "Measurability" OR "Usefulness" | |
| #3 | Quality indicators | | | "Assessment tool*" OR “Benchmarking” OR "Benchmark criteria*" OR “Care quality” OR "Care performance indicator*" OR “Clinical indicators” OR “Evaluation indicator*” OR "Key performance indicator*" OR “Outcome assessment*” OR “Outcome measure*” OR “Performance assessment*” OR “Process Assessment (Health Care)” OR "Performance indicator*" OR "Performance measure*" OR “performance metric*” OR “Quality assessment” OR “Quality assurance*” OR "Quality assessment indicator*" OR "Quality assurance measure*" OR “Quality of care” OR “Quality of health care” OR “Quality of nursing care” OR “Quality indicator*” OR “Quality measure*” OR “Quality metric*” OR "Quality monitoring indicator*" OR “Quality improvement” OR "Quality improvement indicator*” OR "Quality standard*" OR “Resident outcome*” OR “Patient outcome” | |
| Database: Scopus | | | | | |
| Search # | Concept | | | Search Terms | |
| #1 | Aged care centres | | | “Aged care facilit*” OR “Care home*” OR “Elderly care” OR “Elderly home*” OR “Gerontolog* care” OR "Housing for older person*" OR “Home* for the aged” OR “Long term care” OR “Nursing facilit*” OR “Nursing home*” OR “Residential aged care” OR “Residential care” OR “Senior cent*” OR “Senior home*” | |
| #2 | Psychometric properties | | | “Applicab*” OR “Feasib*” OR “Reliab*” OR “Sensitive*” OR “Valid*” | |
| #3 | Quality indicators | | | "Assessment tool*" OR “Benchmarking” OR "Benchmark criteria*" OR “Care quality” OR "Care performance indicator*" OR “Clinical indicators” OR “Evaluation indicator*” OR "Key performance indicator*" OR “Outcome assessment*” OR “Outcome measure*” OR “Performance assessment*” OR “Process Assessment (Health Care)” OR "Performance indicator*" OR "Performance measure*" OR “performance metric*” OR “Quality assessment” OR “Quality assurance*” OR "Quality assessment indicator*" OR "Quality assurance measure*" OR “Quality of care” OR “Quality of health care” OR “Quality of nursing care” OR “Quality indicator*” OR “Quality measure*” OR “Quality metric*” OR "Quality monitoring indicator*" OR “Quality improvement” OR "Quality improvement indicator*” OR "Quality standard*" OR “Resident outcome*” OR “Patient outcome” | |
| Database: Web of Science Core Collection | | | | | |
| Search # | | Concept | | Search Terms | |
| #1 | | Aged care centres | | TS=(“Aged care facilit*” OR “Care home*” OR “Elderly care” OR “Elderly home*” OR “Gerontolog* care” OR "Housing for older person*" OR “Home* for the aged” OR “Long term care” OR “Nursing facilit*” OR “Nursing home*” OR “Residential aged care” OR “Residential care” OR “Senior cent*” OR “Senior home*”) | |
| #2 | | Psychometric properties | | TS=("Applicab*" OR "Feasib*" OR "Reliab*" OR "Sensitive*" OR "Sensitivity" OR "Valid*" OR "Appropriateness" OR "Measurability" OR "Usefulness") | |
| #3 | | Quality indicators | | TS=("Assessment tool*" OR “Benchmarking” OR "Benchmark criteria*" OR “Care quality” OR "Care performance indicator*" OR “Clinical indicators” OR “Evaluation indicator*” OR "Key performance indicator*" OR “Outcome assessment*” OR “Outcome measure*” OR “Performance assessment*” OR “Process Assessment (Health Care)” OR "Performance indicator*" OR "Performance measure*" OR “performance metric** OR “Quality assessment” OR “Quality assurance*” OR "Quality assessment indicator*" OR "Quality assurance measure*" OR “Quality of care” OR “Quality of health care” OR “Quality of nursing care” OR “Quality indicator*” OR “Quality measure*” OR “Quality metric*” OR "Quality monitoring indicator*" OR “Quality improvement” OR "Quality improvement indicator*” OR "Quality standard*" OR “Resident outcome*” OR “Patient outcome”) | |

**Supplementary table 2: Quality assessment according to MMAT**

| **Study No** | **Number of ‘Yes’ answers** | **Number of ‘No’ answers** | **Number of ‘Can’t Tell’ answers** |
| --- | --- | --- | --- |
| 1 | 3 | 2 | 0 |
| 2 | 2 | 1 | 2 |
| 3 | 2 | 2 | 1 |
| 4 | 4 | 0 | 1 |
| 5 | 2 | 1 | 2 |
| 6 | 5 | 0 | 0 |
| 7 | 2 | 1 | 2 |
| 8 | 4 | 1 | 0 |
| 9 | 2 | 3 | 0 |
| 10 | 1 | 1 | 3 |
| 11 | 1 | 2 | 2 |
| 12 | 5 | 0 | 0 |
| 13 | 3 | 2 | 0 |
| 14 | 3 | 1 | 1 |
| 15 | 3 | 1 | 1 |
| 16 | 5 | 0 | 0 |
| 17 | 2 | 3 | 0 |
| 18 | 2 | 3 | 0 |
| 19 | 1 | 2 | 2 |
| 20 | 5 | 0 | 0 |
| 21 | 3 | 1 | 1 |

**Supplementary table 3: Summary of articles included in the systematic review**

| **No** | **Author, Year, and Country** | **QIs (medication -related)** | **Description of QI Numerator (N) and Denominator (D)** | **Psychometric property or other evaluation criteria** |
| --- | --- | --- | --- | --- |
| 1 | Asquier-Khati et al., (2023)  France | Quantity metrics - 14 (Quantity metrics (QMs) measure the volume of antibiotic use) | | -Feasibility  -Validation |
|  |  | Antibiotic consumption | N: Number of antibiotic prescriptions  D: 100 resident-days |  |
|  |  |  | N: DDDs of antibiotics  D: 100 resident-days |  |
|  |  |  | N: Number of residents receiving at least one antibiotic per year  D: Number of residents per year |  |
|  |  | Broad-spectrum antibiotics- co-amoxicillin | N: Number of prescriptions of amoxicillin/clavulanate  D: 100 resident-days |  |
|  |  |  | N: DDDs of amoxicillin/clavulanate  D: 100 resident-days |  |
|  |  | Broad-spectrum antibiotics- cephalosporins | N: Number of prescriptions of cephalosporins  D: 100 resident-days |  |
|  |  |  | N: DDDs of cephalosporins  D: 100 resident-days |  |
|  |  | Broad-spectrum antibiotics- quinolones | N: Number prescriptions of quinolones  D: 100 resident-days |  |
|  |  |  | N: DDDs of quinolones  D: 100 resident-days |  |
|  |  | Second-line antibiotics | N: Number prescriptions of MLSK  D: 100 resident-days |  |
|  |  |  | N: DDDs of MLSK  D: 100 resident-days |  |
|  |  | Route of antibiotic administration | N: Number of prescriptions of parenteral  D: Number of prescriptions of oral + parenteral antibiotics |  |
|  |  | Urine cultures (UC) prescriptions | N: Number of UC/  D: 100 resident-days |  |
|  |  |  | N: Number of residents (regardless their duration of stay) having at least 1 UC per year  D: Number of residents per year |  |
|  |  | Indicators to estimate the appropriateness of antibiotic prescriptions -10 | |  |
|  |  | Antibiotic prescriptions against UTI in men | For male residents only:  N: In the week after UC, number of prescriptions of nitrofurantoin + fosfomycin/trometamol + pivmecillinam + amoxicillin + amoxicillin/clavulanate  D: Number of prescriptions of quinolones + cephalosporins + sulfamethoxazole/trimethoprim |  |
|  |  | Antibiotic prescriptions against UTI in women | For female residents only  N: In the week after UC, number of prescriptions of nitrofurantoin + fosfomycin/trometamol + pivmecillinam D: Number of prescriptions of quinolones for the year |  |
|  |  | Repeated prescription of quinolones | N: Number of prescriptions of quinolones among residents having been prescribed quinolones in the preceding 6 months  D: Number of prescriptions of quinolones for the year |  |
|  |  | Seasonal variation in total antibiotic prescriptions | N: Number of prescriptions of antibiotic during the cold-weather season  D: Number of prescriptions of antibiotic during the hot-weather season −1] × 100 |  |
|  |  | Seasonal variation in quinolones prescriptions | N: Number of prescriptions of amoxicillin/clavulanate during the cold-weather season  D: Number of prescriptions of amoxicillin/clavulanate during the hot-weather season −1] × 100 |  |
|  |  | First-line antibiotics / second-line antibiotics prescriptions | N: Number of prescriptions of amoxicillin + amoxicillin/clavulanate  D: Number of prescriptions of quinolones + cephalosporins + MLSK |  |
|  |  | Duration of antibiotic prescriptions > 7 days | N: Number of prescriptions > 7 days for specific antibiotics  D: Number of antibiotic prescriptions for these antibiotics |  |
|  |  | Co-prescription of antibiotic + systemic NSAIDs | N: Number of antibiotics + systemic NSAID co-prescribed on the same day  D: Number of antibiotic prescriptions |  |
|  |  | Estimated flu vaccine coverage | N: Number of flu vaccines dispensed during the cold-weather season  D: Number of residents during the cold-weather season |  |
|  |  | Route of antibiotic administration | N: Number of prescriptions of oral cephalosporins  D: Number of prescriptions of oral + parenteral cephalosporins |  |
| 2 | Inacio et al., (2023)  Australia | Medication management  – polypharmacy | Percentage of residents prescribed nine or more medications (not including topical, dietary supplements, short term or PRN medications) | Feasibility and Applicability Importance  Appropriate care  Clinical evidence base  Measure specifications |
|  |  | Medication management  – antipsychotics | Percentage of residents who received antipsychotic medications |  |
| 3 | Xu et al., (2023)  USA | Out of 19QIs, the medication-related QI:  Prevalence of antipsychotic medications without a diagnosis of psychosis | N/A | Construct validity  Parsimony and relevance  Usability of QIs to discriminate between facilities  Actionability: Trends in QI rates |
| 4 | Hibbert et al., (2022)  Australia | Medication review | Residents should have a medication review when they:  - have worsening health OR  - have signs of administration problems OR  - are on multiple psychotropic drugs OR  - when a new medicine is ordered | Validity  Feasibility  Acceptability  Impact  Appropriateness |
|  |  |  | Residents and/or family/substitute decision maker should be involved in their medication review. |  |
|  |  | Providing information | Residents and/or family/substitute decision maker who are newly prescribed a medication should be provided with information and education about their medications including:  - consumer medicine information  - current treatment and any changes made  - possible drug-related problems that might occur  - what to do in case side-effects occur or a dose is forgotten  - checking that they understand the treatment and how to implement it. |  |
|  |  | Medication monitoring | Residents who are newly prescribed a medication should receive a monitoring plan. |  |
|  |  | Medication use | Residents prescribed benzodiazepines OR antipsychotics should have a written tapering plan |  |
|  |  |  | Residents with diabetes, who are on insulin therapy or glucose lowering medications should receive a hypoglycaemia management plan. |  |
|  |  |  | Residents who are on psychotropic medications and have fallen should have these reviewed within a week of fall. |  |
| 5 | Jennifer G. Burgess, (2022)  USA | Antipsychotic use | N: Number of patients with any   - antipsychotic use - benzodiazepine use - anxiolytic/sedative/hypnotic use - opioid use - inpatient hospitalisations - antidepressants use - antiepileptic use - memory medication use - non-antipsychotic psychotropic use - emergency department visit   D: Number of patients with dementia in the facility | Feasibility  Importance  Usefulness |
|  |  | Benzodiazepine use |  |  |
|  |  | Anxiolytic/sedative/hypnotic use |  |  |
|  |  | Opioid use |  |  |
|  |  | Inpatient hospitalisation |  |  |
|  |  | Antidepressant use |  |  |
|  |  | Antiepileptic use |  |  |
|  |  | anti-dementia medication use |  |  |
|  |  | Non-antipsychotic psychotropic use |  |  |
|  |  | Emergency Department visit |  |  |
| 6 | Simon et al., (2021)  France | Indicators to estimate the appropriateness of antibiotic prescriptions  Antibiotic prescriptions against UTI in men, ratio | For male residents:  N: Number of prescriptions of nitrofurantoin + certain (fluoro)quinolones [norfloxacin + enoxacin + lomefloxacin + other quinolones + Fosfomycin-trometamol  D: Number of prescriptions of antibiotics for the year | Clinimetric properties:  Applicability  Measurability  Potential room for improvement |
|  |  | Antibiotic prescriptions against UTI in women, ratio | For female residents:  N: Number of prescriptions of nitrofurantoin + pivmecillinam fosfomycin-trometamol  D: Number of prescriptions of quinolones for the year |  |
|  |  | Repeated prescription of quinolones, % | N: number of prescriptions of quinolones among residents having been prescribed quinolones in the preceding 6 months  D: total number of prescriptions of quinolones |  |
|  |  | Seasonal variation in total antibiotic prescriptions, % | [N: Number of prescriptions of antibiotic during the cold-weather season  D: Number of prescriptions of antibiotic during the hot-weather season − 1] × 100 |  |
|  |  | Seasonal variation in quinolone prescriptions, % | [N: Number of prescriptions of quinolones during the cold weather season  D: Number of prescriptions of quinolones during the hot-weather season − 1] × 100 |  |
|  |  | First-line antibiotics/ second-line antibiotics prescriptions, ratio | N: Number of prescriptions of amoxicillin + amoxicillin clavulanate D: Number of prescriptions of quinolones + cephalosporins + MLSK |  |
|  |  | Prescriptions of not indicated antibiotics, % | N: Number of prescriptions of lomefloxacin + moxifloxacin + certain (fluoro)quinolones [norfloxacin + enoxacin + lomefloxacin + other quinolones] + telithromycin + spiramycin-metronidazole + cefaclor + cefadroxil  D: Total number of antibiotic prescriptions |  |
|  |  | Estimated duration of antibiotic prescriptions >8 days, % | N: Number of prescriptions >8 days for specific antibiotics  D: Total number of antibiotic prescriptions for these antibiotics |  |
|  |  | Co-prescription of antibiotic + systemic NSAIDs, % | Number of antibiotics + systemic NSAID(s) co-prescribed on the same day/total number of antibiotic prescriptions |  |
|  |  | Co-prescription of antibiotic + systemic corticosteroids, % | Number of antibiotics + systemic corticosteroids co-prescribed on the same day/total number of antibiotic prescriptions |  |
|  |  | Estimated flu vaccine coverage, % | Number of flu vaccines dispensed during the second semester/ number of residents staying in the NH between October and December |  |
| 7 | Favez et al., (2020)  Switzerland | Polypharmacy: percentage of residents who took 9 or more active ingredients over the last 7 days | N: All residents who had taken 9 or more active ingredients in the last 7 days  D: All long-term care residents | Reliability  Between-provider variability |
| 8 | Mays et al., (2018)  Canada | IF a resident is treated with opioids for pain,  THEN the medical record should document a plan for management of worsening or emergent pain.  IF a resident with persistent pain is treated with opioids,  THEN there should be documentation of a bowel regimen and a plan to document bowel patterns.  IF a resident is started on new opioid therapy for persistent pain,  THEN efficacy and side effects should be assessed within 1 week.  All residents should have a comprehensive medication review with attention to adherence, side-effects, and efficacy within 4 weeks of admission and bi-annually.  IF a resident is prescribed a drug,  THEN the prescribed drug should have an indication or target symptom clearly stated in the medical record or order.  IF a resident is prescribed a new chronic medication,  THEN the subsequent PCP note should document response to therapy and continued need.  IF a resident is prescribed warfarin,  THEN an INR should be determined within 4 d after initiation of therapy and at least every 6 weeks thereafter or there should be documentation that the NHR is being monitored by an  anticoagulation program.  IF a resident is taking warfarin and is prescribed an antibiotic or antiepileptic, THEN the PCP should document a plan for monitoring a follow up INR within 4 days or document that they have notified the NHR’s anticoagulation program of the change.  IF a resident is prescribed an ACE inhibitor or ARB,  THEN he or she should have serum creatinine and potassium monitored within 2 weeks after initiation of therapy and at least yearly thereafter.  IF a resident is prescribed a diuretic or an increased dose of diuretic, THEN he or she should have electrolytes checked within 2 weeks after initiation and at least yearly thereafter.  IF a resident is prescribed a medication with strong anticholinergic effects that is defined by AGS, Beers or STOPP criteria,  THEN there should be documentation of why alternatives were not prescribed.  IF a resident is prescribed a high-risk psychotropic medication such as an antipsychotic, benzodiazepine,  or sedative hypnotic, for insomnia, agitation, or delirium,  THEN there should be documentation that other alternatives including behavioural and environmental interventions have been tried and were not effective.  IF a resident is prescribed a high-risk psychotropic medication such as an antipsychotic, benzodiazepine, or sedative hypnotic,  THEN there should be documentation of ongoing need every 6 months.  IF the PCP plans to discontinue high risk psychotropic medication including benzodiazepines,  sedative hypnotics, antipsychotics,  THEN there should be documentation of gradual dose reduction.  IF a resident is on proton pump inhibitor without a clear indication,  THEN the medication should be discontinued.  IF a resident is prescribed a target-specific anticoagulant (direct oral anticoagulant or novel oral anticoagulant),  THEN laboratory monitoring should include yearly haemoglobin and liver function; as well as on-going renal function measurements (yearly if CrCl >60; every 6 months if CrCl 40e60, or every 3 months if CrCl 15e30).  IF a resident is prescribed a cholinesterase inhibitor,  THEN the perceived cognitive benefits should be evaluated every 6 months.  IF a resident is prescribed a cholinesterase inhibitor,  THEN they should be screened for GI side effects, weight loss, and heart rate assessed within 4 weeks upon admission and every 6 months thereafter.  IF a resident is prescribed a new chronic disease medication,  THEN the following should be noted in the next PCP note:  (i) the indication for the medication  (ii) resident, if able to communicate, was asked about the medication (eg, side effects)  (iii) assessment or plan for follow-up of efficacy or adverse effects of the medication. | | Validity  Feasibility of implementation |
| 9 | Estabrooks et al., (2013)  Canada | Antipsychotic use without psychosis | N/A | Sensitivity to clinical practice |
| 10 | Bell et al., (2012)  Canada | HMG-CoA reductase inhibitors (statins) for all indications | N: Residents who experienced an unintentional discontinuation of their statins/anticoagulant/PPI /thyroxine upon returning to their residence after an acute-care admission  D: residents established as continuous users of statins/anticoagulant/PPI /thyroxine for all indications | Feasibility |
|  |  | Anticoagulants (e.g. warfarin) for the indication of atrial fibrillation |  |  |
|  |  | Proton-pump inhibitors for the indication of post-gastrointestinal haemorrhage |  |  |
|  |  | Thyroxine for all indications |  |  |
| 11 | Courtney et al., (2011)  Australia | Polypharmacy-  (Use of nine or more different medications) | N: Residents who receive nine or more different medications on most recent assessment  D: All residents on most recent assessment. | Face validity  Content validity |
|  |  | 1. No pharmacy review- 2. (Prevalence of medication prescription without pharmacy review) | N: Residents who did not have their medications reviewed by a doctor or pharmacist in the three months prior to the most recent assessment  D: All residents on most recent assessment |  |
|  |  | Prevalence of daily chemical restraints. | N: Residents who are chemically restrained (i.e. through use of psychotropic medication) daily at most recent assessment.  D: All residents at most recent assessment. |  |
| 12 | Jones et al., (2010)  USA | Percent of Residents on antipsychotics without a diagnosis of psychosis | N: Residents receiving antipsychotics on target assessment.  D: All residents with a valid target assessment. | Validity  Reliability |
| 13 | Wenger et al., (2007)  Canada | ACOVE-3 - set of QIs: Domain Medication Use  (24 QIs) | N/A | Validity |
| 14 | Kröger et al., (2007)  Canada | No person with dementia should be taking long-acting sedatives (hypnotics, anxiolytics), unless there is an explicit justification for this medication in the medical record  All vulnerable elders with complex medication regimens who are returning to community living should be evaluated whether they are able to maintain a self-medication program | | Face Validity  Content Validity  Feasibility  Reliability |
| 15 | Hutt et al., (2006)  United States | Pain Medication Appropriateness Scale | The scale has five domains: (1) concordance between pain syndrome and type or class of medication, (2) concordance between dose interval prescribed and half-life of medication, (3) concordance between severity of pain and World Health Organisation level of medication, (4) constipation prevention, and (5) avoidance of geriatric high-risk medications) | Content validity  Construct validity  Interrater reliability  Test retest  reliability |
| 16 | Mor et al., (2003)  USA | Among 22QIs in MDS, medication- related QI was, Days received antipsychotics | N/A | Interrater reliability |
| 17 | Hawes et al., (1997)  USA | Medication use,  antipsychotropic use | N/A | Face validity  Interrater reliability |
| 18 | Morris et al., (1990)  USA | Number of medications | Number of different medications used in the last 7 days | Interrater reliability |
|  |  | New medications | Whether the resident has received new medications during the last 90 days |  |
|  |  | Injections | Number of days injections of any type received during the last 7 days |  |
|  |  | Days received:  -antipsychotics  -antianxiety/hypnotics  -antidepressants | Number of days during the last 7 days where these medications were received |  |
| 19 | Stuijt et al., (2009)  Netherland | Medication Appropriateness Index | | Interrater reliability |
| 20 | Zimmerman et al., (1995) | -Prevalence of antipsychotic use in the absence of psychotic and related conditions  -Prevalence of antipsychotic daily dose in excess of  surveyor guidelines  -Prevalence of antianxiety or hypnotic drug use  -Prevalence of hypnotic drug use on a scheduled or as-needed basis greater than twice in last week  -Prevalence of use of any long-acting benzodiazepine | | Face Validity  Concurrent Validity  Feasibility |
| 21 | Resnick et al (2025)  USA | Appropriate Use  of Opioids | N/A | Validity  Interrater reliability |

CrCl: creatinine clearance; DDD: defined daily dose LTC: long term care, MLSK: macrolide-lincosamide-streptogramin-ketolide, NASID: non-steroidal anti-inflammatory drugs, PCP: primary care providers, PPI: proton pump inhibitor, PRN: pro re nata/as the need arises. UC: urine cultures, UTI: urine track infections

**Supplementary table 4: Additional psychometric properties used in evaluation of QIs in RACHs**

| **QI** | **Psychometric Property** | **Methodologies used to assess the QI of each property** |
| --- | --- | --- |
| 10 medication-related QIs  Jennifer G. Burgess, (2022) | Importance | Delphi Panel with 2 rounds  Participants: n=10, experts included geriatric psychiatry, Gero psychology, geriatric medicine, nursing, pharmacy, and research.  Structured criteria were given,  Participants were asked to rate the measure high on importance if  1. The aspect of care covered by the measure is important to high-quality care for patients with dementia  2. The measure has significance and relevance to stakeholders  3. The measure represents an opportunity for improvement |
| Polypharmacy  Favez et al., (2020) | Between-provider variability | To assess QI’s capacity to distinguish between providers, ICC1, caterpillar plots and rankability computed.  ICC1shows the proportion of variation in the QI that is attributed to the group level. The ICC1 is the ratio of variance among providers (VG) over the total variance (VG+VR). For binary outcomes, VR is the latent scale variance of the logit model π 2 /3. ICC1 values typically range from 0.0 to 0.3, where values over 0.05 indicate relevant between-provider variability.  Caterpillar plots show each nursing home’s estimate for a QI and whether it deviates from the grand mean. They are based on empirical Bayes estimates with 95% confidence intervals.  Rankability measures the part of variability between nursing homes that results from true differences in quality of care. High rankability allows for performance ranking of indicators. Rankability (%) is defined as: % = VG/(VG + median(s^2^)), where median(s^2^) is the variance of the individual facility effect estimates from a fixed effect regression model. Rankability (range: 0–100%) refers to observed differences that might result from quality-of-care disparities and is classed as low (75%). |
| Medication review, Providing information to residents and families, Medication monitoring and Medication use (refer to Supplementary table 3 for more details)  Hibbert et al., (2022) | Impact | A Modified Delphi approach used: The experts scored each indicator using one of three responses (Yes, No and Out of my scope of practice) against indicator impact criteria.   - High impact’ on the resident in terms of domains of quality i.e. safety, effectiveness, resident experience, or access ‘High impact’ within Australian RACF settings e.g. what will be the frequency/ prevalence of presentation   If the indicators had majority score of a ‘No’ across any of the scoring criteria were flagged for exclusion |
| Prevalence of antipsychotic medications without a diagnosis of psychosis  Xu et al., (2023) | Parsimony and relevance | Started with qualitative assessment followed by quantitative assessment. Qualitative assessment of the QIs was conducted that was followed by an empirical analysis. A scoping literature review of NH-QIs and their applications was performed. Expert opinions were requested through four 1-hour meetings with 27 nurses and quality improvement experts from the NH industry, as well as state agency staff that managed the QI system.  Quantitative analysis was done through Pearson correlations for all QIs and scatter plots to identify highly correlated QIs. If a Pearson correlation coefficient between two QIs was near 0.70 or above, and the dots in scatter plots closely clustered on or near a straight line, it was defined as highly correlated and considered combining QIs. Based on the correlation analysis, literature review and expert opinions, decisions were made on combining, dropping, adding or changing the QI definitions. |
|  | Actionability: trends in QI rates | Descriptive analysis (line graphs) was compared trends in the mean QI rate of the best performing 20% of facilities, the median QI rate and the mean QI rate of the worst performing 10% of facilities. If a QI showed substantial improvement resulting in a very low prevalence (eg, median QI rate<1.0%), care quality for a QI may have improved to the point. If performance declined over time or the majority of facilities performed poorly on a QI (eg, median QI rate >50%), then it likely suffers from measurement error or a system-wide failure to address that quality dimension. |
| 11 indicators for the appropriateness of prescription for antibiotics  (refer to Supplementary table 3)  Simon et al., (2021) | Potential room for improvement | This was calculated as 100% minus the performance score (the percentage of NHs that reached the indicator acceptable target). Potential room for improvement was considered insufficient when it was less than 15%.    Note: Indicators were considered having good clinimetric properties when all the 3 of the following criteria were met: measurability of 75% or greater, applicability of 75% or greater, and potential room for improvement of 15% or greater. |
| Percentage of residents prescribed nine or more medications (not including topical, dietary supplements, short term or PRN medications)  Percentage of residents who received antipsychotic medications  Inacio et al., (2023) | Importance | Each indicator was assessed and rated by five of the authors who are researchers and clinicians with significant expertise in the area of quality and safety monitoring and evaluation. For each of the criteria, ratings ranged from whether the indicator does not meet the criteria (1 is the lowest rating) to whether it meets the criteria perfectly (9 is the highest rating). A median score of 1–3 was considered to not meet criteria, a score of 4–6 was considered to meet some criteria and a score of 7–9 was considered to meet criteria.  Criteria for importance  a. Meaningful impact: implementation of this measure will lead to a measurable and meaningful impact.  b. High impact: measure addresses a condition that has a high impact (high prevalence, high morbidity or mortality, high severity of illness and major individual or societal consequences).  c. Performance gap: current performance does not meet best practices and there is opportunity for improvement. |
|  | Appropriate care | Criteria for appropriate care  a. Overuse: measure will promote stopping the use of a test or treatment in individuals in whom the potential harms outweigh the potential benefits.  b. Underuse: measure will encourage use of a test or treatment in individuals in whom the potential benefits outweigh the potential harms.  c. Time interval: time interval to measure the intervention is evidence-based. |
|  | Clinical evidence base | Criteria for clinical evidence base  a. Source: evidence forming the basis of the measure is clearly defined with appropriate references.  b. Evidence: evidence is high quality, high quantity and consistent and represents current clinical knowledge. |
|  | Measure specifications | Criteria for measure specifications  a Clarity: numerator and denominator are clearly defined. This includes outcome measures; numerators detail an outcome that is meaningful to the resident and under the influence of the providers’ care. Denominator includes well-specified and clinically appropriate exceptions to eligibility for the measure.  b Clarity: all components necessary to implement the measure are clearly defined.  c Validity: the measure correctly assesses what it is designed to measure, adequately distinguishing between good and poor quality.  d Reliability: the measure is repeatable and precise, including when data are extracted by different people.  e Risk adjustment: risk adjustment is adequately specified for outcome measures. |

ICC: Intraclass Correlation Coefficient, NH: Nursing Home, QI: Quality Indicator, VG: Group Variance, RV: Residual Variance,
